# Nonlinear Dynamic Trans/Cis Regulatory Circuit for Gene Transcription via Microarray Data

**Published:** 2007-10-12

**Authors:** Yu-Hsiang Chang, Yu-Chao Wang, Bor-Sen Chen

**Affiliations:** Lab of Control and Systems Biology, Department of Electrical Engineering, National Tsing Hua University, Hsinchu, 300, Taiwan

**Keywords:** transcription factor, nonlinear dynamic model, trans/cis regulatory circuit, cell cycle

## Abstract

The trans-regulatory circuit is considered as the regulatory interactions between upstream regulatory genes and transcription factor binding site motifs or cis elements. And the cis-regulatory circuit is viewed as a dynamic interactive circuit among binding site motifs with their effective action on the expression scheme of target gene. In brief, gene transcription depends on the trans/cis regulatory circuits. In this study, nonlinear trans/cis regulatory circuits for gene transcription in yeast are constructed using microarray data, translation time delay, and information of transcription factors (TFs) binding sites. We provide a useful nonlinear dynamic modeling and develop a parameter estimating method for the construction of trans/cis regulatory circuits, which is powerful for understanding gene transcription. We apply our method to construct trans/cis regulatory circuits of yeast cell cycle-related genes and successfully quantify their regulatory abilities and find possible cis-element interactions. Not only could the data of yeast be applied by our method, but those of other species also could. The proposed method can provide a quantitative basis for system analysis of gene circuits, which is potential for gene regulatory circuit design with a desired gene expression.

## Introduction

Due to advances in DNA microarray technology and genome sequencing, it has become possible to measure gene expression levels on a genomic scale. Microarray technology provides insight into the transcriptional state of a cell. In recent studies, the expression profiles and motif binding sites of genes in yeast have been revealed, and regulatory networks have been proposed to explain their regulatory functions (Spellman et al. 1998; Iyer et al. 2001; Simon et al. 2001; Hartemink et al. 2002; Lee et al. 2002; Harbison et al. 2004). However, in order to gain more insight into the infrastructure of the regulatory scheme inside the gene transcription, trans/cis regulatory circuit constructed by the view of the systems biology is an important topic.

**Figure 1:**
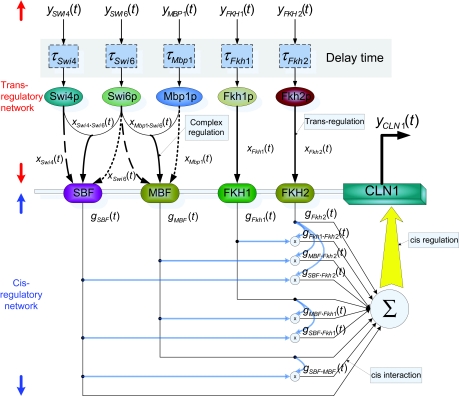
The dynamic trans/cis regulatory model of the gene transcription of target gene CLN1. *x_i_*_·_*_j_* (*t*) denotes trans regulatory function of the complex of the transcription factors i and j and *g_i_*_·_*_j_* (*t*) denotes the interaction between the cis elements i and j.

Genes are always regulated by a number of upstream regulatory genes through their products, for instance, transcription factors bind to specific sites (i.e. cis elements) in the DNA promoter region. The interactions between upstream regulatory genes and cis elements are described by a trans-regulatory circuit (see Fig. 1). The DNA binding motifs, served as anchors to transcription factors, play the role of staying platforms for the assembly of multi-proteins at the output. This mechanism specifies the cis-regulatory circuit, which is viewed as a dynamic interactive circuit among binding site motifs with their effective action on the expression scheme of target gene (Fig. 1). In brief, gene transcription depends on the trans/cis regulatory circuits. Therefore, the comprehension of gene transcriptions needs to recognize the corresponding trans/cis regulatory circuits. Unfortunately, although the cis-regulatory circuit has been widely discussed in *Drosophila* (Berman et al. 2002) and sea urchin (Yuh and Davidson, 1996; Yuh et al. 1996; Yuh et al. 1998), not much is known about trans/cis-regulatory circuits because of their complicated interactive schemes, which are not easily detected directly by conventional experiments. That is why we want to propose a method to construct trans/cis regulatory circuits to understand gene transcription process thoroughly.

Given a processed data set, one expects to be ready to tackle the biological data interpretation problem. It is popular to use clustering, classification, and projection methods to analyze the data set. However, most analysis methods only use microarray data, not both dynamic expression time profiles of microarray data and the information of motif binding sites (Gardner et al. 2003; Chen et al. 2004; Chen et al. 2005). Furthermore, the interactions among proteins and delay from transcription to translation are not considered in their models of gene transcription. Recently, systems biology and computational biology methods have been widely considered to describe the biological functions from the dynamic system perspective (Yeung et al. 2002; Davidson et al. 2003; Tegner et al. 2003; Chen et al. 2004; Sontag et al. 2004; Chang et al. 2006). Engineering theory has also been used to know more about biological complexity (Carlson and Doyle, 1999; Yi et al. 2000; Csete and Doyle, 2002; Hasty et al. 2002; Gardner et al. 2003; Tegner et al. 2003). Here, we provide a nonlinear dynamic model to gain more insight into trans/cis regulatory circuits for gene transcription using the knowledge of microarray data, the information of motif binding sites, translation delays and protein complexes from the systems biology perspective.

In this study, a nonlinear dynamic model is proposed to describe the kinetics of trans/cis regulatory circuits for gene transcriptions of yeast genes. In the trans circuit part (see Fig. 1), the transcription regulatory functions on cis elements are described by the regulatory activities of transcription factors and reaction of complexes among transcription factors. The binding from upstream regulatory genes to the transcription factor binding sites is described by a biological sigmoid function to model the binding (activating the binding function) and no binding (inactivating the binding function) signals beyond or below some threshold of mRNA concentrations of regulatory genes. The translation time delay from the mRNA of a regulatory gene to transcription factor is also considered in our model. The reactions (cooperations) of complexes of transcription factors are represented by nonlinear interactions (Chang et al. 2006). In this study, nonlinear interactions between two proteins and among three proteins are also considered to mimic the effect of protein complexes on a gene transcription regulation process. In the cis-regulatory circuit part, the interactions of cis elements are also modeled by a nonlinear dynamic equation. The regulatory functions of double and triple nonlinear interactions among the cis elements are considered in this dynamic model. Furthermore, the decay rate is also considered to describe how the output mRNA is degraded in the target gene.

The nonlinear dynamic model is useful to describe how the upstream regulatory genes control a target gene to produce the output expression of mRNA through its trans/cis regulatory circuit. Using the information on transcription factors (Simon et al. 2001) and the experimental profiles on upstream regulatory genes and target gene (Spellman et al. 1998), the model of trans/cis regulatory circuit of target gene can be transformed to an algebraic regression equation for parameter estimation of the dynamic transcription model. With this study, the stochastic noises of microarray data are also considered to describe the uncertainties of the data. This is capable of improving the accuracy of parameter estimations in the dynamic model. The kinetic coefficients of the stochastic dynamic model, the decay rate of target gene’s mRNA and the variance of noises are estimated via the maximum likelihood estimation method and the downhill simplex search method. After estimating these parameters via expression profiles of the target gene and its regulatory genes, they will be substituted into the nonlinear dynamic model to confirm the validity of trans/cis regulatory circuits. For illustration, the trans/cis regulatory circuits of the gene CLN1 are constructed in detail by the proposed method from expression profiles in the microarray data (Spellman et al. 1998) and knowledge of binding site motifs of yeast cell cycle (Simon et al. 2001). After constructing the model, we are able to use the mRNA expression profiles of regulatory genes to predict the expression profiles of the target gene and analyze the characteristics of the nonlinear trans/cis regulatory circuits to gain more insight into gene transcription, which also provides a potential method for gene regulatory circuit design with a desired gene expression.

## Modeling and Identification

First of all, we construct a dynamic trans/cis regulatory circuit with two parts. One simulates the binding effects of regulatory proteins and their complexes, and the other considers interactions of cis elements in the target gene. After that, we apply the downhill simplex search method and the maximum likelihood method to estimate the dynamic parameters of the trans/cis regulatory circuit. Finally, we show the experimental data that have been used and how to preprocess it before system identification of regulatory circuits. The proposed method will provide a way for system analysis of gene regulatory circuits and a method for gene circuit design with a desired gene expression.

### Dynamic model of trans/cis regulatory circuits

First, we consider a trans/cis regulatory network as a system block with several regulatory genes as inputs and a target gene as output. In our model, we use the mRNA expression data of upstream regulatory genes corresponding to transcription factors as the system input and the mRNA expression data of the target gene as the system output. For the illustration of nonlinear dynamic trans/cis regulatory circuit, an example of the target gene CLN1 is shown in Figure 1. From the binding site motif data (Simon et al. 2001), we know that the gene CLN1 has five transcription factors (Swi4, Swi6, Mbp1, Fkh1, and Fkh2). Some of the investigations (Koch et al. 1993; Uetz et al. 2000; Ito et al. 2001) show that some proteins could form a protein complex via protein-protein interaction, and then the protein complex binds to the motif of the target gene. The binding of transcription factors on binding site motifs is described by the sigmoid function to model the binding (ON) and nonbinding (OFF) through some threshold (Goldbeter and Koshland, 1981; Mestl et al. 1995; Gardner et al. 2003; Klipp et al. 2005). In the trans-regulatory circuit, we also consider this protein complex effect. The translated proteins Swi4 and Swi6 will form a protein complex SBF as a transcription factor to bind to the motif in the target gene, and proteins Mbp1 and Swi6 form another complex MBF (Iyer et al. 2001). Therefore, in Figure 1, we use linear functions and nonlinear functions to express the trans-regulatory functions of the transcription factors and their complexes, respectively. The trans-regulatory functions *g_SBF_* (*t*), *g_MBF_* (*t*), *g_Fkh_*_1_ (*t*), and *g_Fkh_*_2_ (*t*) of the respective cis elements SBF, MBF, Fkh1, and Fkh2 are the regulatory result of the expression profiles of transcription factors and their complexes interactions. In the trans-regulatory circuit, these trans-regulatory functions on cis elements, which are generated from upstream transcription factors and their interactive complexes, are shown as follows

(1) $$\begin{array}{l} g_{SBF}(t)=a_{Swi4}x_{Swi4}(t)+a_{Swi6}x_{Swi6}(t)\\ +a_{Swi4\cdot Swi6}x_{Swi4\cdot Swi6}(t)\\ g_{MBF}(t)=a_{Mbp1}x_{Mbp1}(t)+a_{Swi6}x_{Swi6}(t)\\ +a_{Mbp1\cdot Swi6}x_{Mbp1\cdot Swi6}(t)\\ g_{Fkh1}(t)=a_{Fkh1}x_{Fkh1}(t)\\ g_{Fkh2}(t)=a_{Fkh2}x_{Fkh2}(t) \end{array}$$

where *g_i_* (*t*) denotes the trans-regulatory function to the cis element *i*, *x_i_* (*t*) denotes the reaction of the transcription factor *i* on the binding site, *x_i_*_·_*_j_* (*t*) denotes the complex interaction between transcription factors *x_i_* (*t*) and *x_j_* (*t*), *a_i_* denotes the regulatory ability of the transcription factor *x_i_* (*t*) to the cis elements, and *a_i_*_·_*_j_* denotes the regulatory ability of the complex due to transcription factors *x_i_* and *x_j_*.

After considering the trans effects of regulatory proteins and their protein complexes on cis elements, we also take into account the cis effects of regulatory interactions of cis elements on the transcription of coding region of the target gene. We suppose that the interaction function of two cis elements could be represented by the product of trans-regulatory functions from their transcription factors. From Figure 1, the interactions of cis elements are represented as follows:

(2) $$\begin{array}{l} g_{SBF\cdot MBF}(t)=b_{SBF\cdot MBF}x_{Swi4}(t)\cdot x_{Swi6}(t)\cdot x_{Mbp1}(t)\\ g_{SBF\cdot Fkh1}(t)=b_{SBF\cdot Fkh1}x_{Swi4}(t)\cdot x_{Swi6}(t)\cdot x_{Fkh1}(t)\\ g_{SBF\cdot Fkh2}(t)=b_{SBF\cdot Fkh2}x_{Swi4}(t)\cdot x_{Swi6}(t)\cdot x_{Fkh2}(t)\\ g_{MBF\cdot Fkh1}(t)=b_{MBF\cdot Fkh1}x_{Swi6}(t)\cdot x_{Mbp1}(t)\cdot x_{Fkh1}(t)\\ g_{MBF\cdot Fkh2}(t)=b_{MBF\cdot Fkh2}x_{Swi6}(t)\cdot x_{Mbp1}(t)\cdot x_{Fkh2}(t)\\ g_{Fkh1\cdot Fkh2}(t)=b_{Fkh1\cdot Fkh2}x_{Fkh1}(t)\cdot x_{Fkh2}(t) \end{array}$$

where *g_i_*_·_*_j_* (*t*) denotes the interaction between cis elements *i* and *j*. The coefficient *b_i_*_·_*_j_* denotes the corresponding interactive ability.

The trans-regulatory functions in Equation (1) and their cis interactions in Equation (2) will lead to the transcription of gene CLN1 with mRNA expression profiles *y_CLN_*_1_(*t*) in Figure 1. The transcriptional behavior of cis regulatory circuit will be described by the following stochastic dynamic equation.

(3) $${\dot{y}}_{CLN1}(t)=G(t)-\lambda y_{CLN1}(t)+ɛ(t)$$

where the nonlinear transcriptional regulatory function *G*(*t*) is denoted as

$$\begin{array}{l} G(t)=g_{SBF}(t)+g_{MBF}(t)+g_{Fkh1}(t)+g_{Fkh2}(t)\\ +g_{SBF\cdot MBF}(t)+g_{SBF\cdot Fkh1}(t)+g_{SBF\cdot Fkh2}(t)\\ +g_{MBF\cdot Fkh1}(t)+g_{MBF\cdot Fkh2}(t)+g_{Fkh1\cdot Fkh2}(t)+c \end{array}$$

and *λ* denotes the decay rate of mRNA expression profiles, which represent the degradation of mRNA. The constant *c* denotes the basal level of regulation, which comes from other factors than transcription factors. The gene regulatory function *G*(*t*) in (3) denotes the whole transcriptional regulation from the cis elements due to the binding of transcription factors of the gene CLN1 and the basal level from other factors. ɛ(*t*) denotes a stochastic noise due to the uncertainty and the fluctuation of mRNA microarray data.

The biological meaning of Equation (3) is that the change of mRNA level in the gene CLN1 is the synthesis due to the transcriptional regulatory function *G*(*t*) and the degradation −*λy*_CLN1_(*t*). The mRNA expression of the gene CLN1 will increase if the synthesis regulatory function *G*(*t*) is greater than the degradation *λy*_CLN1_(*t*). Otherwise, it will decrease. Substituting Equations (1) and (2) into Equation (3), we get the dynamic equation of trans/cis regulatory circuit in the gene transcription processing of the gene CLN1 as follows

(4) $$\begin{array}{l} {\dot{y}}_{CLN1}(t)=a_{Swi4}x_{Swi4}(t)+2a_{Swi6}x_{Swi6}(t)+a_{Mbp1}x_{Mbp1}(t)\\ +a_{Fkh1}x_{Fkh1}(t)+a_{Fkh2}x_{Fkh2}(t)\\ +a_{Swi4\cdot Swi6}x_{Swi4\cdot Swi6}(t)+a_{Mbp1\cdot Swi6}x_{Mbp1\cdot Swi6}(t)\\ +b_{SBF\cdot MBF}x_{Swi4}(t)\cdot x_{Swi6}(t)\cdot x_{Mbp1}(t)\\ +b_{SBF\cdot Fkh1}x_{Swi4}(t)\cdot x_{Swi6}(t)\cdot x_{Fkh1}(t)\\ +b_{SBF\cdot Fkh2}x_{Swi4}(t)\cdot x_{Swi6}(t)\cdot x_{Fkh2}(t)\\ +b_{MBF\cdot Fkh1}x_{Swi6}(t)\cdot x_{Mbp1}(t)\cdot x_{Fkh1}(t)\\ +b_{MBF\cdot Fkh2}x_{Swi6}(t)\cdot x_{Mbp1}(t)\cdot x_{Fkh2}(t)\\ +b_{Fkh1\cdot Fkh2}x_{Fkh1}(t)\cdot x_{Fkh2}(t)\\ +c-\lambda y_{CLN1}(1)+ɛ(t) \end{array}$$

In the above dynamic equation of gene transcription, *x_i_* (*t*) denotes the expression profiles of the binding transcription factors. The nonlinear term $\prod_{i}x_{i}(t)$ denotes the interactions of transcription factors in the trans/cis regulatory circuit. The coefficients in the dynamic gene circuit denote the corresponding regulatory abilities of the corresponding regulatory functions and will be identified later by the corresponding microarray data.

However, at present, it is still not easy to measure directly the expression profiles of transcription factors *x_i_* (*t*) in Equation (4). Because the expression profiles of yeast mRNA are available, in this study, the expression levels of these transcription factors will be replaced by expression levels of mRNA microarray data of their upstream regulatory genes but with some translation process delay in the cell. Now, for system identification of trans/cis regulatory circuit, it is more practical to consider the biochemical reactive relation between the transcription factor profiles *x_i_* (*t*) at the motif binding sites and their relevant mRNA expression profiles *y_i_* (*t*) of the upstream regulatory gene (Goldbeter and Koshland, 1981; Mestl et al. 1995). For this purpose, we describe the binding reaction function *x_i_* (*t*) of the transcription factor on its motif binding site as a sigmoid function of mRNA expression profiles of the corresponding regulatory gene (Klipp et al. 2005). Further, it takes time for mRNA to translate into proteins as transcription factors and move to motif binding sites of the following target gene (Arava et al. 2003). We should consider this translation delay time to the sigmoid binding function when using mRNA expression profiles *y_i_* (*t*) to replace expression levels *x_i_* (*t*) of transcription factors in the trans/cis dynamic model (Table 1).

(5) $$x_{i}(t)=f_{i}(y_{i}(t-\tau_{i}))=\frac{1}{1+\text{exp}\left\{-r\left[y_{i}(t-\tau_{i})-M_{i}\right]\right\}}$$

where *r* denotes the transition rate of the sigmoid function, *M_i_* denotes the mean of mRNA expression level of the regulatory gene *i*, and τ*_i_* denotes the time delay of the translation time from mRNA to transcription factor (protein) for the regulatory gene *i*. In Figure 1, *y_i_* (*t*) and *x_i_* (*t*) represent the mRNA expression profile and the binding regulation function of the corresponding transcription factor on its motif binding site, respectively. The biochemical meaning of Equation (5) is that the regulation of transcription factor *x_i_* (*t*) on the binding site motifs is between ON (binding) and OFF (no binding) signal with some transition region dependent on beyond or below some threshold of mRNA expression level of the regulatory gene after a time delay τ*_i_*, which is available in Arava et al. (Arava et al. 2003). The sigmoid function can also be considered as a method to normalize the expression profiles of regulatory genes between 0 and 1 to model the binding and no binding, which has been successfully employed to describe the binding of regulatory gene (Chen et al. 2004; Klipp et al. 2005; Chang et al. 2006).

Hence, based on the mRNA expression time profiles and the translation delay time, the dynamic equation of trans/cis regulatory circuit for the gene transcription of CLN1 is described by

(6) $$\begin{array}{l} {\dot{y}}_{CLN1}(t)\\ =a_{Swi4}f_{Swi4}(y_{Swi4}(t-\tau_{Swi4}))\\ +2a_{Swi6}f_{Swi6}(y_{Swi6}(t-\tau_{Swi6}))\\ +a_{Mbp1}f_{Mbp1}(y_{Mbp1}(t-\tau_{Mpb1}))\\ +a_{Fkh1}f_{Fkh1}(y_{Fkh1}(t-\tau_{Fkh1}))\\ +a_{Fkh2}f_{Fkh2}(y_{Fkh2}(t-\tau_{Fkh2}))\\ +a_{Swi4\cdot Swi6}f_{Swi4\cdot Swi6}(y_{Swi4}(t-\tau_{Swi4})\cdot y_{Swi6}(t-\tau_{Swi6}))\\ +a_{Mbp1\cdot Swi6}f_{Mbp1\cdot Swi6}(y_{Mbp1}(t-\tau_{Mbp1})\cdot y_{Swi6}(t-\tau_{Swi6}))\\ +b_{SBF\cdot MBF}f_{Swi4}(y_{Swi4}(t-\tau_{Swi4}))\cdot f_{Swi6}(y_{Swi6}(t-\tau_{Swi6}))\\ \cdot f_{Mbp1}(y_{Mbp1}(t-\tau_{Mbp1}))\\ +b_{SBF\cdot Fkh1}f_{Swi4}(y_{Swi4}(t-\tau_{Swi4}))\cdot f_{Swi6}(y_{Swi6}(t-\tau_{Swi6}))\\ \cdot f_{Fkh11}(y_{Fkh1}(t-\tau_{Fkh1}))\\ +b_{SBF\cdot Fkh2}f_{Swi4}(y_{Swi4}(t-\tau_{Swi4}))\cdot f_{Swi6}(y_{Swi6}(t-\tau_{Swi6}))\\ \cdot f_{Fkh12}(y_{Fkh2}(t-\tau_{Fkh2}))\\ +b_{MBF\cdot Fkh1}f_{Swi6}(y_{Swi6}(t-\tau_{Swi6}))\cdot f_{Mbp1}(y_{Mbp1}(t-\tau_{Mbp1}))\\ \cdot f_{Fkh11}(y_{Fkh1}(t-\tau_{Fkh1}))\\ +b_{MBF\cdot Fkh2}f_{Swi6}(y_{Swi6}(t-\tau_{Swi6}))\cdot f_{Mbp1}(y_{Mbp1}(t-\tau_{Mbp1}))\\ \cdot f_{Fkh2}(y_{Fkh2}(t-\tau_{Fkh2}))\\ +b_{Fkh1\cdot Fkh2}f_{Fkh1}(y_{Fkh1}(t-\tau_{Fkh1}))\cdot f_{Fkh2}(y_{Fkh2}(t-\tau_{Fkh2}))\\ +c-\lambda y_{CLN1}(t)+ɛ(t) \end{array}$$

**Figure 2:**
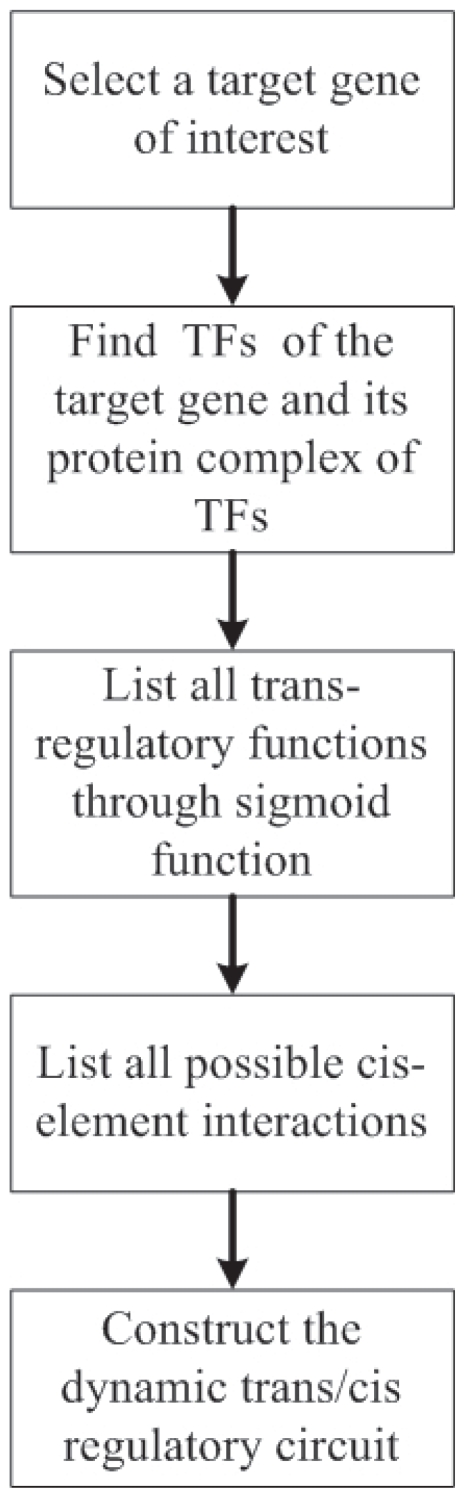
Block diagram to construct a dynamic trans/cis regulatory circuit for gene transcription.

Based on the above analysis, in general, a block diagram to construct trans/cis regulatory circuit of gene transcription is shown in Figure 2, and the nonlinear dynamic model of trans/cis regulatory circuit of gene transcription of any gene of interest in yeast can be described as follows

(7) $$\begin{array}{l} \dot{y}(t)=\sum_{i=1}^{N}a_{i}f_{i}(y_{i}(t-\tau_{i}))+\sum_{i,j}a_{i\cdot j}f_{i\cdot j}(y_{i}(t-\tau_{i})\cdot y_{j}(t-\tau_{j}))\\ +\sum_{i,j,k}a_{i\cdot j\cdot k}f_{i\cdot j\cdot k}(y_{i}(t-\tau_{i})\cdot y_{j}(t-\tau_{j})\cdot y_{k}(t-\tau_{k}))+\cdots \\ +\sum_{p}\left[b_{p\cdot q}\prod_{r}f_{i}(y_{i}(t-\tau_{i}))\right]+c-\lambda y(t)+ɛ(t) \end{array}$$

Unlike the previous dynamic gene regulatory models only with linear regulatory terms (Gardner et al. 2003; Chen et al. 2004; Chen et al. 2005), in our model, nonlinear interactions among two proteins and three proteins are also considered in our gene expression dynamic model. Furthermore, the delay from transcription to translation is also considered in our dynamic model. According to the information on protein complexes and motif binding sites in the promoter region of the gene, *y*(*t*) denotes the mRNA expression profiles of a gene of interest and *y_i_*(*t*), *i* ∈ {1 2 … *N*}, denote the mRNA expression profiles of upstream regulatory genes. With sampling expression in the next section, Equation (7) can be rewritten as.

(8) $$\dot{y}(t)+\lambda y(t)=\sum_{i=1}^{L}\theta_{i}\xi_{i}(t)+c+ɛ(t)$$

where ξ*_i_*(*t*) *i* ∈ {1 2 … *L*} denote all possible regulatory functions, *θ_i_* *i* ∈{1 2 … *L*} denote the corresponding regulatory abilities (coefficients), and *L* is the total number of input functions.

The next step is to identify the parameters *θ_i_*, *λ*, *c*, and the covariance *σ*^2^ of noise from mRNA microarray data. The parameter estimations of *θ_i_*, *λ*, *c*, and *σ*^2^ are achieved by the combination of downhill simplex search algorithm and maximum likelihood parameter method using the Methods in the sequel. After the parameter estimation is finished, we could construct a trans/cis regulatory circuit for any gene of interest via mRNA microarray data and the motif binding site information of transcription factors and their complexes.

### Experimental data

We use the yeast microarray hybridization data of Spellman et al. (Spellman et al. 1998) as our mRNA expression profiles. They have many experimental methods to reset the yeast cell cycle to measure mRNA expression profiles for the whole genome comprehensively. Here, we use the experimental cell cycle data of the “*α* factor”. The information of motif binding sites in a target gene is from Simon et al. (Simon et al. 2001), Lee et al. (Lee et al. 2002) and Harbison et al. (Harbison et al. 2004), and then the protein complex data are obtained from Simon et al. (Simon et al. 2001) and the MIPS database. In the study of Simon et al. (Simon et al. 2001), there are 9 major transcription factors of cell cycle genes in yeast, so we use these 9 major transcription factors as the regulatory input of our dynamic model, which could also avoid overfitting in parameter estimation due to only 18 time points in expression profiles. Using the above information, we could construct dynamic models as Equation (7) for cell cycle genes and then identify the parameters from mRNA microarray data by the proposed parameter estimation algorithm methods.

We also include the translation delay time to our dynamic model. The data of translation delay time is available from Arava et al. (Arava et al. 2003).

## Results

### Reconstruction of microarray raw data by the gene regulatory circuits

We use the “*α* factor” microarray data of Spellman et al. (Spellman et al. 1998) and information on 9 major transcription factors from Simon et al. (Simon et al. 2001) to construct our nonlinear dynamic trans/cis model. In the study of Simon et al. (Simon et al. 2001), there are 769 cell cycle genes, but only 189 cell cycle genes have at least one motif binding site of 9 major transcription factors (P-value < 0.0015). Therefore, we could construct dynamic trans/cis regulatory circuits for these 189 cell cycle genes.

**Figure 3:**
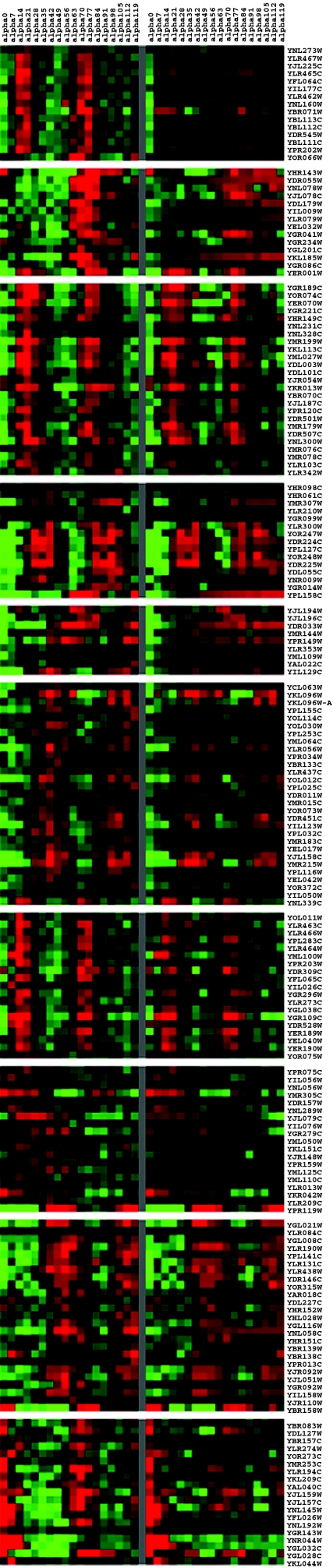
Comparison between the experimental mRNA expression profiles and those predicted by the proposed model. The experimental mRNA expression profiles of 189 cell cycle genes are at left side, and the profiles which are generated by the predicted dynamic regulatory circuits are at right side. Genes represented by red tonalities are over expressed and those represented by green ones are down regulated. The correlation coefficient of both profiles is 0.7276.

**Figure 4:**
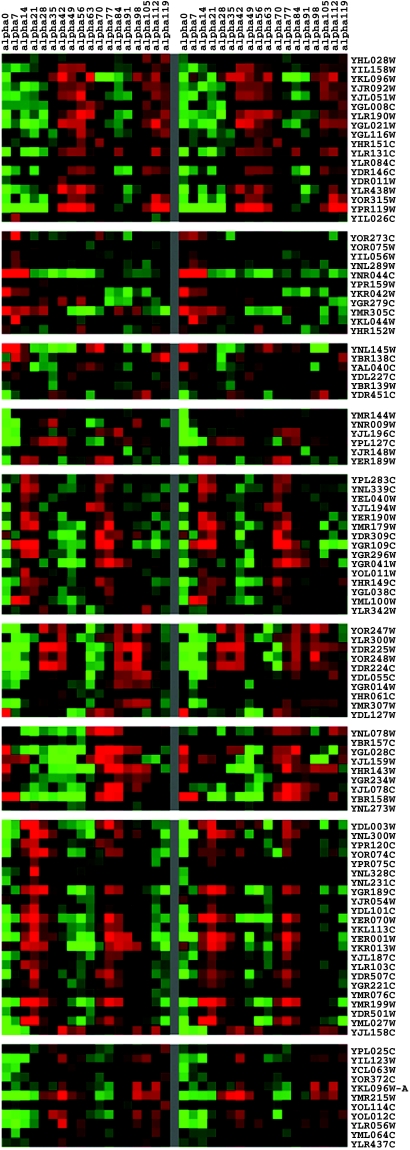
Comparison between the experimental mRNA expression profiles and those predicted by the proposed model. The experimental mRNA expression profiles of 109 cell cycle genes are at the left side, and the profiles predicted by the dynamic regulatory circuits are at the right side. And the correlation coefficient of both profiles is 0.8502.

After estimating the parameters of dynamic trans/cis regulatory circuit for each gene, we compare the actual expression profiles with the constructed expression profiles of 189 genes by the cluster analysis and visualization tool (details can be found in Methods). The comparison can be seen in Figure 3 and we the correlation coefficient of these two data is 0.7276. However, we found that 80 cell cycle genes have only one motif binding site of 9 major TFs. These genes may have other motif binding sites which are not of the 9 major transcription factors, or they have post-transcription to dominate their expression profiles. For example, FUN26, SHL7, STE2, AGA2, YBL111C, YBL112C, and YBL113C have many other motif binding sites, which are not in the 9 major transcription factors (Lee et al. 2002; Harbison et al. 2004). Thus these 9 transcription factors may not play dominating roles in these genes, and the performances of predicted expression profiles of these genes by dynamic trans/cis regulatory circuits may not be very satisfactory. After removing 80 genes with one motif binding site from 189 genes, the dynamic trans/cis regulatory circuits of 109 cell cycle genes with at least two motif binding sites of these 9 transcription factors are considered to be more accurate. The actual profiles and the predicted profiles by the dynamic trans/cis regulatory circuits are shown in Figure 4, and their correlation coefficient is 0.8502. We also use the chi-square method to test the hypothesis of trans/cis circuit by comparing our predicted data and real microarray data. We found that 176 of 189 target genes cannot be rejected by the proposed trans/cis circuit by 95% significance, and other 13 target genes cannot be tested by the chi-square method because the number of their parameters is more than the number of their expression profiles data.

### Test of shuffled microarray data

In order to test the accuracy of our model, we shuffled Spellman et al.’s “α factor” microarray data (Spellman et al. 1998) of expression profiles by random. After shuffling the microarray data, we applied our method to construct another new trans/cis regulatory circuit and then used the new regulatory circuit to generate profiles. For the 189 target genes with at least one motif of 9 major TFs, the correlation coefficient between shuffled profiles and their corresponding profiles predicted by new regulatory circuits via shuffled data is 0.1143 (shown in supplementary Figure S1), but the correlation coefficient between actual profiles and their corresponding predicted profiles is 0.7276. Furthermore, for the 109 target genes with at least two more motifs of 9 major TFs, the correlation coefficient between shuffled profiles and their corresponding profiles predicted by shuffled data is 0.5939 (shown in supplementary Figure S2), but the correlation coefficient between actual profiles and their corresponding predicted profiles is 0.8502. Obviously, only data generated by real biological systems could be identified well by the proposed model. From the view of system identification, a proper system model will lead to a good system identification if overfitting could be avoided. In our case, the proposed model is applicable to the trans/cis regulatory circuit because the correlation coefficient of real microarray data is much larger than that of shuffled microarray data. So we can say that our dynamic model is proper for the trans/cis regulatory circuit of gene expression program of yeast.

### Regulatory activities of TFs in the target gene

From the estimated parameters, we can find which transcription factor activates or represses its target genes and quantify its activity. Therefore, we can see which transcription factor can affect certain target gene a lot, and why the same transcription factor may cause different effects on different target genes. In Table 2, we can see there are many transcription factors regulating the target gene CLN1. CLN1, a G1/S-specific cyclin, is regulated by SBF, MBF, Fkh1, and Fkh2 (Simon et al. 2001). By the estimated kinetic parameters of MBF (Swi6, Mbp1, and Mbp1 · Swi6), we found that Swi6 dominates the effect of MBF on target gene CLN1, and it may play an important regulatory role in CLN1. The kinetic parameters of Mbp1 and Mbp1 · Swi6 are of smaller scale, which means that Mbp1 may play a certain kind of role but not an important regulatory role in CLN1. Mbp1 may be necessary for CLN1, but has no more affection on CLN1 after the expression of Mbp1 exceeds certain level. This result could reflect that Mbp1 is a DNA-binding component of MBF, and Swi6 may have a regulatory function (Iyer et al. 2001). For parameters of SBF (Swi4, Swi6, and Swi4 · Swi6), Swi4 and Swi6 play major roles of SBF in CLN1, but the role of Swi4 · Swi6 is minor. It shows that the linear combination of Swi4 and Swi6 has significant effect on target gene CLN1. Hence, we consider that Swi6 may have a regulatory function of SBF in CLN1, and Swi4 is also significant on target gene CLN1. From Table 2, Fkh1 and Fkh2 contribute negative regulation to target gene CLN1. Comparing these kinetic parameters, we know that Fkh1, Swi4, and Swi6 affect target gene CLN1 more than Fkh2 and Mbp1 do.

Our model also provides another important estimation of interactions among the cis elements. All possible interactions among the cis elements are considered, and then their kinetic parameters are estimated by expression profiles of microarray data. These estimated kinetic parameters could not only tell us the possibilities of these interactions of cis elements, but also the significance of these interactions. For example, we consider 5 cis-element interactions in target gene CLN1. From the kinetic parameters estimated, there are three parameters larger than the others. We may conclude that these three terms, MBF · Fkh2, SBF · Fkh1, and SBF · Fkh2, could be the three possible interactions among real cis elements.

With the same transcription factor binding to different target genes, different kinetic parameters of this transcription factor determine different regulatory effects on the target genes. A larger parameter of the same transcription factor binding to different target genes means that it plays a more important role and is more sensitive than those binding to another target gene. From our estimated kinetic parameters, we can compare these effects on different target genes with the same transcription factor. The G_1_ cyclin PCL2, which associates with Pho85p cyclin-dependent kinase (Cdk) to contribute to entry into the mitotic cell cycle, is regulated by SBF, Ace2, and Swi5. (Simon et al. 2001). On the kinetic parameters of PCL2 (see Table 2), the effect of transcription factor SBF on PCL2 is dominated by Swi6 and Swi4 · Swi6. For another target gene MNN1, required for addition of alpha1,3-mannose linkages to N-linked and O-linked oligosaccharides, the effect of transcription factor SBF on MNN1 is also dominated by Swi6 and Swi4 · Swi6, which can be seen in SGD database. However, the kinetic parameters of SBF on PCL2 (Simon et al. 2001) are all smaller than those of SBF on MNN1. We can consider that SBF has more regulatory effect on MNN1 than PCL2 and the sensitivity of SBF on MNN1 is more than that on PCL2. After simulation by changing the expression profiles of Swi4 or Swi6, of which SBF is composed, we find that the result is the same as we discussed above.

### Possibilities of cis-element interactions

In order to find the most possible cis-element interactions, we sorted all possible kinetic parameters of cis-element interactions calculated by our method as a distribution and then found those within two-sided 90% confidence interval of the distribution. There are 314 possible cis-element interactions within the 189 cell cycle genes selected before, and then we found 15 cis-element interaction terms in each side of this two-sided interval. Some of these 30 interaction terms appear more frequently, which include complex interactions of Swi4/Mbp1/Swi6, Fkh2/Swi4/Swi6, and Ace2/Swi5. In Table 3, we count the frequencies of these cis-element interactions, which occur more than two times within the two-sided 90% confidence interval.

For the complex interactions of Swi4/Mbp1/Swi6, both SBF, the complex of Swi4 and Swi6, and MBF, the complex of Mbp1 and Swi6, control the transcription of G1/S cyclin genes, and many genes, Cdc6, Swi4, Clb6, Swe1, and Cln1, are both bound by SBF and MBF at the same time. Therefore, it is possible that the activators (Swi4/Mbp1/Swi6) have a cis-element interaction, and the possibility of their relationship has been reported by Kato et al. (Kato et al. 2004). The other two possible cis-element interaction terms (Fkh2/Swi4/Swi6 and Ace2/Swi5) calculated by our method may also exist. A few genomic analyses have indicated the involvement of SBF and Fkh1/Fkh2 in S phase (Lee et al. 2002), and SBF and Fkh2 most probably regulate budding, cell-wall synthesis, and spindle-related genes in S phase (Kato et al. 2004). Ace2 and Swi5 are a pair of TFs of yeast that regulate the expression of many cell cycle-specific genes, including Sic1, an inhibitor of Cdc28 protein kinase, Rme1, a regulator of meiosis, and Ash1, a regulator of meiosis (Doolin et al. 2001). In recent studies, Ace2 and Swi5 cooperate to induce the expressions of a subset of genes, but the antagonistic interaction of Ace2 and Swi5 was found (Doolin et al. 2001). With 82% identical DNA binding domains, Ace2 and Swi5 bind to the same DNA sequence (McBride et al. 1999), and it is possible that proteins compete for access on these promoters, but only one activates transcription (Doolin et al. 2001). Therefore, one partner of Swi5 and Ace2 sometimes can have a stronger contribution towards regulation, and the antagonistic interaction of Ace2 and Swi5 found is not surprising.

## Discussion

Our method uses mRNA expression profiles, protein complexes, translation time delay, and the information of binding site motif to construct a dynamic trans/cis regulatory circuit to gain more insight into the gene expression of yeast. It is more precise to use these kinds of data to construct a nonlinear stochastic regulatory model for the gene transcription. Furthermore, a stochastic regulatory model can easily describe the properties of change, interaction, and uncertainty in mRNA expression profiles. Recently, Vu and Vohradsky also used nonlinear dynamic model to infer the transcriptional regulators of target genes (Vu and Vohradsky, 2007). They successfully identified possible transcriptional factors of yeast cell cycle, which shows the power of nonlinear dynamic models.

Constructing the trans/cis regulatory circuits of the cell cycle genes of yeast is useful to quantify the influence of transcription factors on their target genes, and then the possibilities of cis element interactions could be found from the statistical perspective. The confirmation of these possible cis element interactions would be a direction of further research. In addition, the proposed method can provide a quantitative basis for system analysis of gene circuit and give a scheme for gene circuit design with a desired gene expression in the future. Not only could the data of *Saccharomyces cerevisiae* be applied by our method, but those of other species also could. Any species with their mRNA expression profiles and the information of binding site motif can be applied using this method to construct their trans/cis regulatory circuits.

However, in this study, we found that the mRNA expression profiles of several genes generated by the predicted dynamic trans/cis regulatory circuit have a little difference from their original mRNA expression profiles. It may be because 9 transcriptional factors are not enough in these cases in which the genes may not be controlled dominantly by 9 TFs. We may use ChIP-chip data (Harbison et al. 2004) to replace the 9 major TFs from Simon et al. (Simon et al. 2001) to construct the transcriptional regulatory circuit. 204 DNA-binding transcriptional factors are found in yeast (Harbison et al. 2004), which may be useful for this elaboration of the transcriptional regulatory circuit. Furthermore, only 18 time points of mRNA expression profiles are used to estimate the parameters of dynamic model in each gene, which are not enough to get precise parameter estimation because of the large number of kinetic parameters to be estimated, which will easily lead to overfitting. If we include all possible 204 TFs into our model, the number of parameters that we want to estimate may be so large that over-fitting would happen. Consequently, we only choose the most important 9 TFs in our model to avoid the overfitting in parameter estimation. If there are more time point profiles of mRNA expression and information of binding site motifs, the accuracy of the dynamic trans/cis regulatory circuit could be improved by the proposed method.

In conclusion, unlike the convention linear dynamic models, this study provides a nonlinear stochastic dynamic trans/cis regulatory circuit for any species via mRNA expression profiles, the information of binding site motif, protein complexes, and translation time delay to gain more insight into transcriptional regulatory infrastructures of gene expression program of yeast. The results are confirmed by a statistical hypothesis test.

## Methods

### Parameter estimation of dynamic trans/cis regulatory circuit

The dynamic trans/cis regulatory circuit of a gene of interest can be described by the dynamic Equation (7) according to the mRNA expression profiles of upstream regulatory genes and their nonlinear interactions. The parameters of the dynamic trans/cis circuit are then specified to meet the practical input/output microarray data of the regulatory genes and the target gene.

In order to avoid the interruption of high frequency noise with the use of the derivative information *ẏ*(*t*), the solution of the dynamic Equation (8) is expressed by the following integration equation.

(9) $$\begin{array}{l} y(t)=y(0)\cdot e^{-\lambda t}+\sum_{i}\theta_{i}e^{-\lambda t}\int_{0}^{t}e^{\lambda \tau }\xi_{i}(\tau )d\tau +ce^{-\lambda t}\int_{0}^{t}e^{\lambda \tau }d\tau \\ +e^{-\lambda t}\int_{0}^{t}ɛ(\tau )e^{\lambda \tau }d\tau \\ =\text{K}e^{-\lambda t}+\sum_{i}\theta_{i}e^{-\lambda t}\int_{0}^{t}e^{\lambda \tau }\xi_{i}(\tau )d\tau +k+e(t) \end{array}$$

where $k\equiv \frac{c}{\lambda }$ and $\text{K}\equiv y(0)-\frac{c}{\lambda }$ are constants. Noise of *y*(*t*) is written with *e*(*t*) ≡ *e*^−^*^λt^*∫_0_*^t^ɛ*(*t*)*e^λτ^d*, i.e. the mRNA concentration could be generated by dynamic Equation (9) through upstream regulatory mRNA *y*_1_(*t*) *y*_2_ (*t*) … *y_N_* (*t*) if their parameters could be estimated.

In Equation (9), ξ*_i_* (*t*) is a combinative regulatory function of the regulatory genes. We use a third order polynomial cubic spline method to approximate ξ*_i_* (*t*) through microarray data with 18 time points, and then use the partial integration method to calculate *e*^−^*^λt^*∫_0_*^t^e*^λτ^ξ*_i_* (τ)*d*τ (Faires and Burden, 1998).

For ξ*_i_* (*t*) function, we use the cubic spline method to approach it. Cubic spline is a method of using a third order polynomial to approach every four data points (Faires and Burden, 1998). So we can rewrite the function ξ*_i_* (*t*) as follows.

(10) $$\xi_{i}(t)=\alpha_{i}t^{3}+\beta_{i}t^{2}+\gamma_{i}t+\delta_{i}$$

From Equations (9) and (10), we can use partial integration to refine Equation (9) as follows.

(11) $$\begin{array}{l} y(t)=\Lambda e^{-\lambda t}+\sum_{i}\theta_{i}\cdot \left(\frac{\xi_{i}(t)}{\lambda }-\frac{\xi_{i}^{\prime }(t)}{\lambda^{2}}+\frac{\xi_{i}^{\prime \prime }(t)}{\lambda^{3}}-\frac{\xi_{i}^{\prime \prime \prime }(t)}{\lambda^{4}}\right)\\ +k+e(t) \end{array}$$

where ξ*_i_*′(*t*) = 3*α_i_t*^2^ + 2 *β_i_t* + *γ_i_* ξ*_i_*″(*t*) = 6*α_i_t* + 2*β_i_* and ξ*_i_*‴(*t*) = 12*α_i_* and

$$\Lambda =\text{K}-\sum_{i}\theta_{i}\cdot \left(\frac{\xi_{i}(0)}{\lambda }-\frac{{\xi_{i}}^{\prime }(0)}{\lambda^{2}}+\frac{{\xi_{i}}^{\prime \prime }(0)}{\lambda^{3}}-\frac{{\xi_{i}}^{\prime \prime \prime }(0)}{\lambda^{4}}\right).$$

After applying the cubic spline method to solve and rewrite the dynamic Equation (9), we use a search algorithm, downhill simplex search method, for parameter estimations (Jang et al. 1997). However, this search method is not very efficient. Therefore, we combine the downhill simplex search method and maximum likelihood method to estimate the parameters. We use the downhill simplex search method to estimate the nonlinear parameter *λ* at first and then use the maximum likelihood method to estimate linear parameters K, *θ_i_*, and *k*.

After estimating *λ* by downhill simplex research method, we use the method of maximum likelihood to estimate the other linear parameters. Equation (11) can be written as the following regression form.

(12) $$\begin{array}{l} y(t)=[e^{-\lambda t}\left(\frac{\xi_{1}(t)}{\lambda }-\frac{\xi_{1}^{\prime }(t)}{\lambda^{2}}+\frac{\xi_{1}^{\prime \prime }(t)}{\lambda^{3}}-\frac{\xi_{1}^{\prime \prime \prime }(t)}{\lambda^{4}}\right)\\ \cdots \left(\frac{\xi_{i}(t)}{\lambda }-\frac{\xi_{i}^{\prime }(t)}{\lambda^{2}}+\frac{\xi_{i}^{\prime \prime }(t)}{\lambda^{3}}-\frac{\xi_{i}^{\prime \prime \prime }(t)}{\lambda^{4}}\right)\;1]\left[\begin{array}{c} \Lambda \\ \theta_{1}\\ \vdots \\ \theta_{i}\\ k \end{array}\right]+e(t)\\ \equiv \varphi (t)\cdot \theta +e(t) \end{array}$$

where *φ*(*t*) denotes the regression vector which can be obtained from the above processing. *θ* is the parameter vector of trans/cis regulatory circuit which is to be estimated to make the transcriptional expression of trans/cis circuit meet the microarray data.

If we have a lot of microarray data points to process by the method above to get values of

$$\left\{y(t_{l})\left(\frac{\xi_{l}(t)}{\lambda }-\frac{\xi_{l}^{\prime }(t)}{\lambda^{2}}+\frac{\xi_{l}^{\prime \prime }(t)}{\lambda^{3}}-\frac{\xi_{l}^{\prime \prime \prime }(t)}{\lambda^{4}}\right)\right\}.$$

for *l* ∈ {12 … 18} and, *i* ∈ {12 … *L*} the parameter vector can be estimated by the following approach. Using a matrix notation, Equation (12) at different time points is of the following form

(13) $$\left[\begin{array}{c} y(t_{1})\\ y(t_{2})\\ \vdots \\ y(t_{18}) \end{array}\right]=\left[\begin{array}{c} \varphi (t_{1})\\ \varphi (t_{2})\\ \vdots \\ \varphi (t_{18}) \end{array}\right]\cdot \theta +\left[\begin{array}{c} e(t_{1})\\ e(t_{2})\\ \vdots \\ e(t_{18}) \end{array}\right]$$

For simplicity, we can further define the notations *Y*, *Φ*, and E to represent Equation (13) as follows.

(14) Y=Φ·θ+E

In Equation (13), we assume noises *e*(*t_i_*) at different time points as independent random variables of normal distribution with zero mean and unknown variance *σ*^2^, i.e. the variance of E is ∑ = *E*{EE*^T^*} = *σ*^2^*I*, where *I* is a unit matrix. In this study, a maximum likelihood parameter estimation method will be used to estimate *θ* and *σ*^2^ from microarray data of regulatory genes and the target gene (Johansson, 1993). If E is assumed to be normally distributed with N elements, its probability density function is of the following from

(15) $$p(\text{E})=\frac{1}{{\left({\left(2\pi \right)}^{N}\text{det}\Sigma \right)}^{-1/2}}\text{exp}\left\{-\frac{{\text{E}}^{T}\Sigma^{-1}\text{E}}{2}\right\}$$

Under Equation (14) we have the likelihood function

(16) $$p(\theta ,\sigma^{2})=\frac{1}{{\left(2\pi \sigma^{2}\right)}^{-N/2}}\text{exp}\left\{-\frac{{\left(Y-\Phi \cdot \theta \right)}^{T}(Y-\Phi \cdot \theta )}{2\sigma^{2}}\right\}$$

Equation (16) can be considered as a function of parameters *θ* and *σ*^2^. We want to specify *θ* and *σ*^2^ to maximize the likelihood function in (16). It is practical to take the logarithm of their likelihood function, and then we have the following log-likelihood function as follows.

(17) $$\text{log}L(\theta ,\sigma^{2})=-\frac{N}{2}\text{log}(2\pi \sigma^{2})-\frac{1}{2\sigma^{2}}\sum_{k=1}^{N}{\left[y(t_{k})-\varphi (t_{k})\cdot \theta \right]}^{2}$$

where *y*(*t_k_*) and *φ*(*t_k_*) are the k-th elements of *Y* and *Φ*, respectively.

Here we expect the log-likelihood function to have the maximum at *θ* = *θ̂* and *σ̂*^2^ and *σ̂*^2^. The necessary condition for maximum likelihood estimates *θ̂* and *σ̂*^2^ as follows (Johansson, 1993)

(18) $$\begin{array}{l} {\frac{\partial \text{log}L(\theta ,\sigma^{2})}{\partial \theta }|}_{\theta =\hat{\theta }}=0\\ {\frac{\partial \text{log}L(\theta ,\sigma^{2})}{\partial \sigma^{2}}|}_{\sigma ={\hat{\sigma }}^{2}}=0 \end{array}$$

The estimated parameters *θ̂* and *σ̂*^2^ are shown below.

(19) $$\hat{\theta }={\left(\Phi^{T}\Phi \right)}^{-1}\Phi^{T}Y$$

(20) $$\begin{array}{l} {\hat{\sigma }}^{2}=\frac{1}{N}\sum_{k=1}^{N}\left[y(t_{k})-\varphi (t_{k})\cdot \hat{\theta }\right]\\ =\frac{1}{N}{\left(Y-\Phi \cdot \hat{\theta }\right)}^{T}(Y-\Phi \cdot \hat{\theta }) \end{array}$$

*N* where *Y* and *Φ* can be obtained from the microarray data of regulatory genes and the target gene.

**Figure 5:**
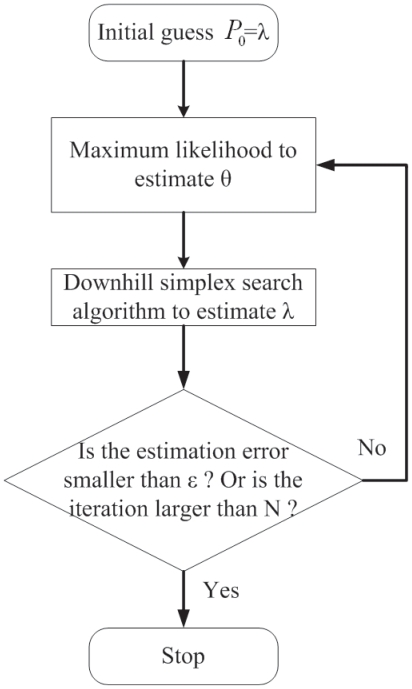
The combined method of the downhill simplex search and the maximum likelihood estimation for *λ* and *θ* iteratively.

After estimating *θ̂* by maximum likelihood method, we take *θ̂* into downhill simplex search method to estimate *λ* again. We iterate these two methods until the differences of real data and estimated data are as small as possible. All iteration processes are shown in Figure. 5.

### Comparison between actual/constructed expression profiles

We use the cluster analysis and visualization tool (Cluster and Treeview) written by Michael Eisen (http://rana.lbl.gov/EisenSoftware.htm) to compare the actual expression profiles with the constructed expression profiles. When clustering the expression profiles, we use hierarchical clustering methods (Eisen et al. 1998).

## Supplementary materials

Figure S1 Comparison between the shuffled experimental mRNA expression profiles and those predicted by the proposed model. The shuffled experimental mRNA expression profiles of 189 cell cycle genes are at the left side, and the profiles predicted by the dynamic regulatory circuits are at the right side. And the correlation coefficient of both profiles is 0.1143.

Figure S2 Comparison between the shuffled experimental mRNA expression profiles and those predicted by the proposed model. The shuffled experimental mRNA expression profiles of 109 cell cycle genes are at the left side, and the profiles predicted by the dynamic regulatory circuits are at the right side. And the correlation coefficient of both profiles is 0.5939.

## Figures and Tables

**Table 1:** Translation delay time of 9 major transcription factors.

Transcription factor	Translation delay time(min)
Fkh1	unavailable
Fkh2	1.056818384
Mcm1	0.050641356
Ace2	0.20647784
Swi5	0.223001589
Ndd1	0.638633059
Swi4	0.495504558
Mbp1	0.408482192
Swi6	0.71182446

**Table 2:** Parameters of dynamic trans/cis regulatory models of CLN1, TR2, and MNN1 based on Equation (4).

CLN1	Terms due to MBF	−0.009*x*Mbp1(*t* − 0.4) + 0.025*x_Swi_*_6_(*t* − 0.7) − 0.0027 *f_Mbp_*_1·_*_Swi_*_6_(*y_Mbp_*_1_(*t* − 0.4) · *y_Swi_*_6_(*t* − 0.7))
*ẏ*_CLN1_(*t*)=	Terms due to SBF	−0.01*_Swi_*_4_(*t* − 0.5) + 0.02516*x_Swi_*_6_(*t* − 0.7) + 0.003435 *f_Swi_*_4·_*_Swi_*_6_(*y_Swi_*_4_(*t* − 0.5) · *y_Swi_*_6_(*t* − 0.6))
	Terms of Fkh1 and Fkh2	−0.36396*x_Fkh_*_1_(*t*) − 0.024962*x_Fkh_*_2_ (*t* − 1)
	Interaction between MBF and SBF	−0.0027745*x_Swi_*_4_(*t* − 0.5)*x_Swi_*_6_ (*t* − 0.7) *x_Mbp_*_1_(*t* − 0.4)
	Interaction between SBF and Fkh1	−0.011154*x_Swi_*_4_ (*t* − 0.5)*x_Swi_*_6_ (*t* − 0.7)*x_Fkh_*_1_ (*t*)
	Interaction between SBF and Fkh2	−0.014026*x_Swi_*_4_(*t* − 0.5)*x_Swi_*_6_(*t* − 0.7)*x_Fkh_*_2_ (*t*− 1)
	Interaction between MBF and Fkh1	−0.0057164*x_Swi_*_6_ (*t*− 0.7)*x_Mbp_*_1_ (*t* − 0.4)*x_Fkh_*_1_(*t*)
	Interaction between MBF and Fkh2	−0.046408*x_Swi_*_6_ (*t* − 0.7) *x_Mbp_*_1_(*t* − 0.4)*x_Fkh_*_2_ (*t* − 1)
	Interaction between Fkh1 and Fkh2	−0.0023141*x_Fkh_*_1_ (*t*)*x_Fkh_*_2_(*t*− 1)
	Decay rate and basal level	−4.0839 − 0.20719*y_CLN_*_1_(*t*)
PCL2	Terms due to SBF	0.0167*x_Swi_*_4_(*t* − 0.5) − 0.0384*x_Swi_*_6_ (*t* − 0.7) − 0.0464 *f_Swi_*_4·_*_Swi_*_6_ (*y_Swi_*_4_(*t* − 0.5) · *y_Swi_*_6_(*t*− 0.6))
*ẏ*_PCL2_(*t*)=	Terms due to Ace2 and Swi5	+0.070897*x_Ace_*_2_(*t* − 0.21) + 0.1044*x_Swi_*_5_(*t*− 0.22)
	Interaction between Ace2 and Swi5	+0.045873*x_Ace_*_2_(*t* − 0.21)*x_Swi_*_5_ (*t*− 0.22)
	Interaction between SBF and Ace2	+0.17035*x_Swi_*_4_(*t* − 0.5)*x_Swi_*_6_ (*t* − 0.7)*x_Ace_*_2_(*t* − 0.21)
	Interaction between SBF and Swi5	+0. 093195*x_Swi_*_4_(*t*− 0.5)*x_Swi_*_6_ (*t*− 0.7)*x_Swi_*_5_(*t* − 0.22)
	Decay rate and basal level	+1. 2225 − 0.43022 *y_PCL_*_2_(*t*)
MNN1	Terms due to SBF	−0.0432*x_Swi_*_4_ (*t* − 0.5) − 0.394*x_Swi_*_6_(*t* − 0.7) − 0.142 *f_Swi_*_4·_*_Swi_*_6_ (*y_Swi_*_4_(*t* − 0.5) · *y_Swi_*_6_(*t* − 0.6))
*ẏ*_MNN1_(*t*)=	Decay rate and basal level	+0.81055 − 0.45975*y_MNN_*_1_(*t*)

**Table 3:** The possible cis element interactions which appear within the two-sided 90% confidence interval (there are 30 cis-element interactions within the two-sided 90% confidence interval).

Possible cis element interactions	Counts of appearances within the two-sided 90% confidence interval
Swi4/Mbp1/Swi6	5
Fkh2/Swi4/Swi6	4
Ace2/Swi5	3
Mcm1/Swi5	3
Fkh1/Fkh2	2
Fkh2/Mcm1	2
Fkh2/Mbp1/Swi6	2
Mcm1/Swi4/Swi6	2
Swi4/Swi5/Swi6	2
