# Supplementary figures and images for: Nonlinear Dynamic Trans/Cis Regulatory Circuit for Gene Transcription via Microarray Data
Source: Gene Regul Syst Bio. 2007 Oct 12;1:151–66. (PMC2759131)

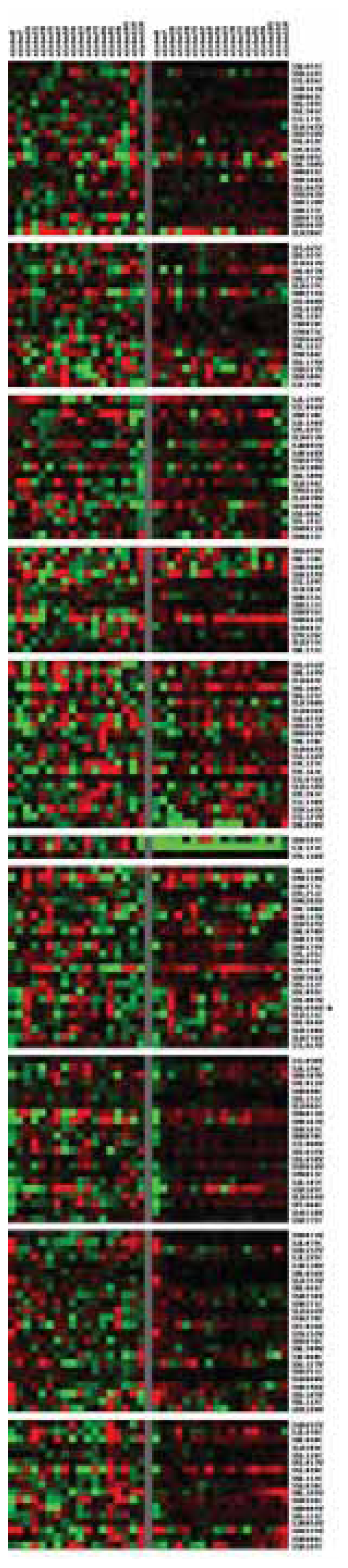

Supplement: Figure S1 — Comparison between the shuffled experimental mRNA expression profiles and those predicted by the proposed model. The shuffled experimental mRNA expression profiles of 189 cell cycle genes are at the left side, and the profiles predicted by the dynamic regulatory circuits are at the right side. And the correlation coefficient of both profiles is 0.1143. [file grsb-2007-151f6.tif]

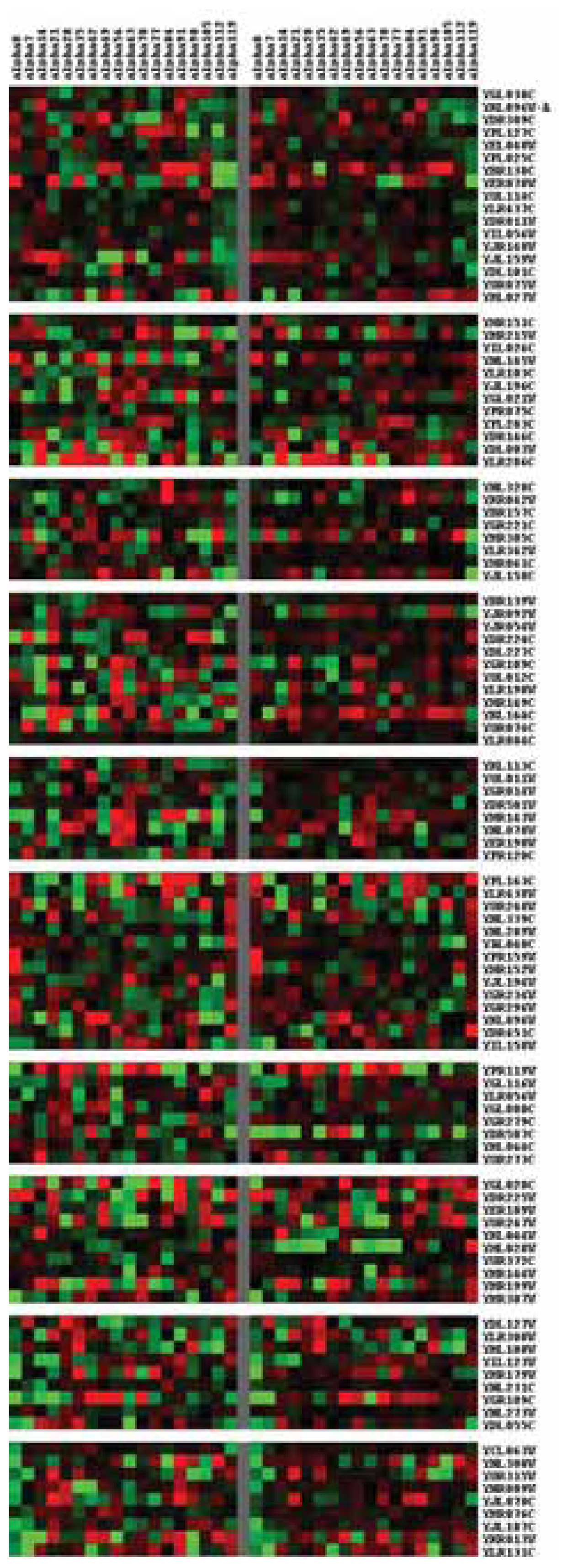

Supplement: Figure S2 — Comparison between the shuffled experimental mRNA expression profiles and those predicted by the proposed model. The shuffled experimental mRNA expression profiles of 109 cell cycle genes are at the left side, and the profiles predicted by the dynamic regulatory circuits are at the right side. And the correlation coefficient of both profiles is 0.5939. [file grsb-2007-151f7.tif]
